# Conservation Genomics of *Apocynum venetum*: Genetic Adaptation and Demographic History Across China's Saline‐Alkali Ecosystems

**DOI:** 10.1111/eva.70191

**Published:** 2025-12-28

**Authors:** Na Yuan, Chunlin Jia, Ruisen Lu, Xingxing Yuan, Xin Chen

**Affiliations:** ^1^ Institute of Industrial Crops, Jiangsu Academy of Agricultural Sciences Nanjing China; ^2^ Institute of Leisure Agriculture, Shandong Academy of Agricultural Sciences Jinan China; ^3^ Jiangsu Key Laboratory for Conservation and Utilization of Plant Resources Institute of Botany, Jiangsu Province and Chinese Academy of Sciences Nanjing China

**Keywords:** *Apocynum venetum*, genome assembly, genome‐environment association, population genomics

## Abstract

*Apocynum venetum* L., a saline‐alkali‐tolerant plant valued for its high‐quality bast fiber in textile manufacturing and medicinal compounds in traditional medicine, serves as a key economic species in saline‐alkali regions with additional phytoremediation applications. However, its natural populations are becoming increasingly threatened by rapid environmental change and anthropogenic activities. To inform conservation and sustainable utilization, we generated a chromosome‐level genome assembly of *A. venetum* (234.73 Mb; contig N50 = 19.11 Mb, scaffold N50 = 20.46 Mb) using PacBio HiFi, Illumina and Hi‐C technologies, and performed whole‐genome resequencing of 109 individuals spanning China's saline‐alkali regions. Population genetic analyses revealed that the Xinjiang population exhibited the highest level of genetic diversity and strong genetic differentiation from the other populations. Demographic analyses indicated that most populations underwent significant population declines during the late Last Glacial Maximum, followed by recovery in western and northern populations, whereas the eastern coastal populations maintained consistently low effective population sizes. Genome‐environment association analyses identified candidate adaptive loci, including a *flavonol 4′‐sulfotransferase* (*4′‐ST*) gene, potentially linked to saline‐alkali tolerance and flavonoid biosynthesis. Our findings provide critical insights into the evolutionary history and adaptive mechanisms of *A. venetum*, offering genomic tools for conservation prioritization and the development of stress‐resilient cultivars through marker‐assisted breeding.

## 1 Introduction

Plants adapted to saline and alkaline conditions have evolved a series of special survival strategies, making them valuable resources for the phytoremediation of salt‐affected soils (Rao et al. 2023). In China, saline‐alkaline land covers approximately 10% of the country's total land area and hosts over 500 species of natural saline‐alkali‐tolerant plants, including *Apocynum venetum* L., a species with substantial economic potential due to its applications in medicine, textiles, and ecological restoration (Zhao et al. 2011; Li et al. 2014). Despite its ecological and industrial importance, wild populations of *A. venetum* are increasingly threatened by climate change and anthropogenic activities, leading to genetic erosion and heightened extinction risks (Newbold et al. 2015; Pinto et al. 2024). Therefore, understanding the genetic diversity and adaptive mechanisms of *A. venetum* is critical for its conservation and sustainable utilization.

*A. venetum* is a deciduous subshrub widely distributed across saline‐alkali soils and arid desert regions of China (Zhang et al. 2006). This species exhibits remarkable ecological adaptability with a strong tolerance to salinity, drought, and high temperatures. It also has significant economic value as a medicinal herb and a source of textile materials. Since the 1950s, extensive research has focused on its biological characteristics, textiles, chemical composition, and pharmacological properties (Xiang et al. 2023). However, the genetic basis of its stress tolerance and economically important traits remains poorly understood. Previous studies using RAPD markers on nine *A. venetum* populations revealed generally low genetic diversity, with northwestern arid populations showing higher diversity than northeastern ones, though no clear geographic genetic structure was detected (Peng et al. 2008). Subsequent RAPD and ISSR analyses demonstrated significant correlations between genetic variation and environmental factors, with Xinjiang populations demonstrating negative correlations between genetic diversity and both precipitation and altitude, whereas populations from Inner Mongolia, Gansu, and Jilin showed negative correlations between genetic diversity and mean annual temperature (Su 2013; Gen 2021). Recent genomic advances, including a draft genome assembly (Li et al. 2019) and a chromosome‐scale reference (Dorjee et al. 2024), have laid the groundwork for linking genetic variation to key agronomic and industrial traits. However, the genome assemblies are based on materials from the northwestern region, limiting their representativeness. Furthermore, previous population genetic studies, while broader in scope, have relied on a limited number of molecular markers, which may not fully capture the species' genetic diversity and evolutionary history. Importantly, the genetic mechanisms underlying its adaptation to saline‐alkaline environments, a key trait for breeding improved varieties, have yet to be systematically investigated.

Local adaptation is typically studied through reciprocal transplantation and garden experiments (Anderson et al. 2011). However, the application of these approaches is often limited in wild species with long generation times and difficulties in measuring fitness‐related traits (Neale and Kremer 2011). In recent years, the integration of population genomic data with association analysis methods, such as genotype‐environment association (GEA), has emerged as a powerful strategy for studying local adaptation in nonmodel plant species (Lasky et al. 2023; Feng et al. 2024). For example, based on whole‐genome resequencing data from 874 individuals of European beech ( *Fagus sylvatica* L.), GEA analyses identified key adaptive variants, including a candidate gene for winter temperature adaptation (Lazic et al. 2024). Similarly, GEA analyses of *Achnatherum splendens* based on high‐quality transcriptome data revealed functional divergence in duplicated genes under salt stress, providing valuable insights into evolutionary mechanisms in saline habitats (Ren et al. 2022). These findings underscore the utility of GEA in uncovering fine‐scale patterns of local adaptation and enhancing our understanding of plant responses to environmental pressures, thereby providing conservation and breeding strategies for climate resilience.

In this study, we generated a chromosome‐level genome assembly for *A. venetum* by integrating PacBio HiFi, Illumina, and Hi‐C technologies and resequenced the genomes of 109 individuals across its distribution range. Using these genomic resources, we aimed to: (1) elucidate the species' population structure and evolutionary history, informing conservation strategies; and (2) identify genomic regions underlying local adaptation, particularly those linked to salinity tolerance, fiber quality, and medicinal compound biosynthesis, with direct implications for agricultural and industrial utilization. By integrating genomic and environmental data, this study not only advances our understanding of *A. venetum*'s adaptive evolution but also provides actionable insights for its commercial cultivation and genetic improvement in saline‐alkaline regions.

## 2 Methods

### 2.1 Plant Materials and Genome Sequencing

**FIGURE 1:**
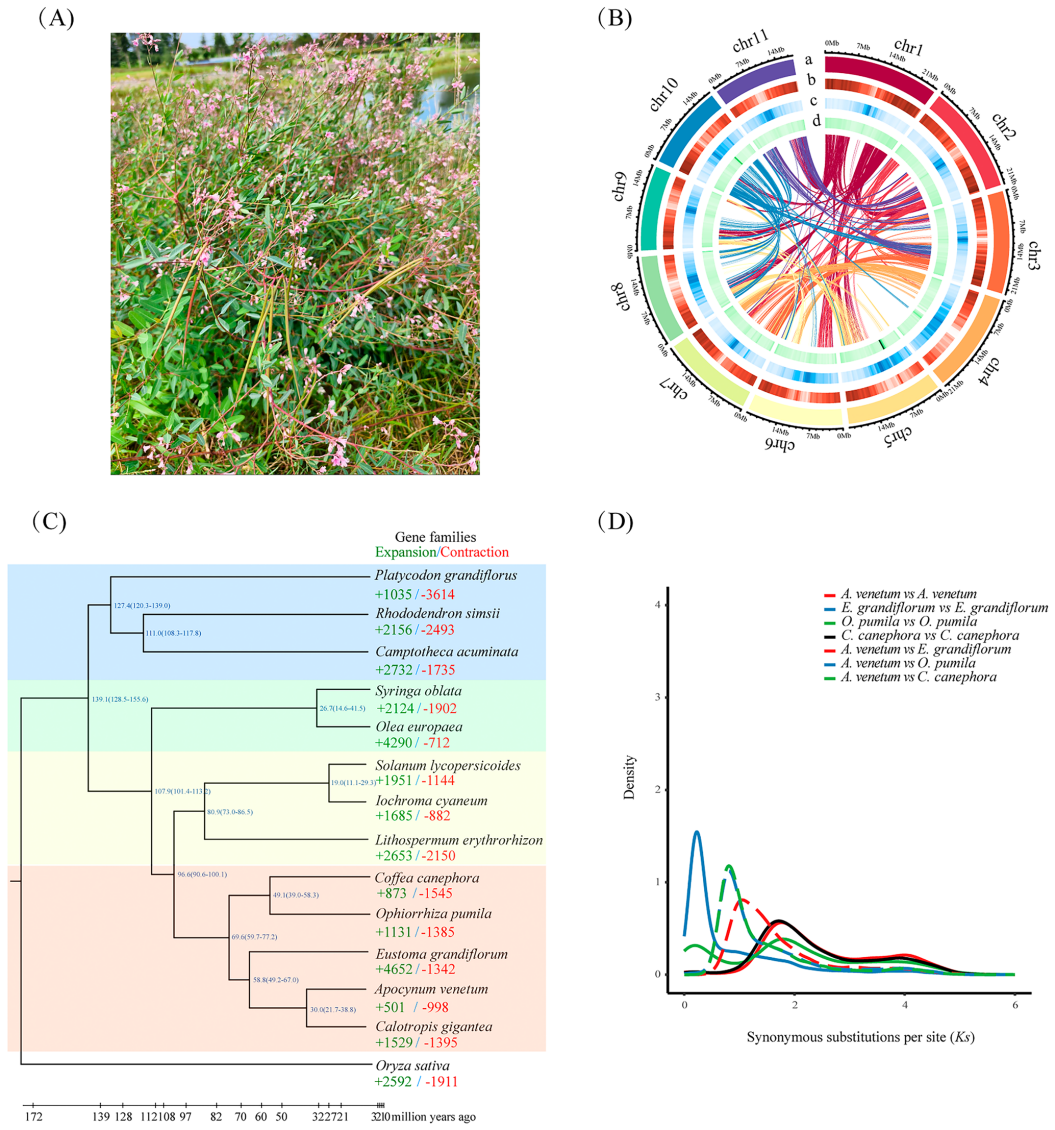
Morphology and genome evolution of *A. venetum*. (A) Photo of wild *A. venetum*. (B) Circos display of the genome characteristics. Tracks from outermost to innermost are (a) the 11 chromosomes at the Mb scale gene distribution, (b) gene density, (c) distribution of repeat elements and (d) GC content of the genome. Center is intra‐genome collinear blocks connected by curved lines. (C) Phylogenetic relationships, divergence time and expansion and contraction of gene families of *A. venetum* and other 13 plant species. The estimated divergence time (million years ago) is labelled at each branch site. Green and red numbers represent the expanded or contracted gene families, respectively. (D) Distribution of average synonymous substitution levels (*Ks*) between syntenic blocks.

Fresh leaves from an individual wild specimen *A. venetum* collected in Yancheng, Jiangsu Province, China (33°28′37.92″ N, 120°36′27″ E) were used for genomic DNA extraction (Figure 1A). This location was identified using a herbarium specimen (ReferenceVoucher: PE 01952832) as a geographical reference. High‐quality genomic DNA was extracted using a modified CTAB method (Lutz et al. 2011). Illumina‐compatible sequencing libraries were constructed from qualified genomic DNA using the Nextera DNA Flex Library Prep Kit (Illumina, USA). Briefly, the DNA was randomly sheared, end‐repaired, A‐tailed, and adapter‐ligated. The final libraries, with an insert size of ~350 bp, were quality‐controlled using Qubit 2.0 and Agilent 2100 Bioanalyzer and quantified via qPCR. Paired‐end sequencing was performed on an Illumina NovaSeq 6000 platform (Illumina, San Diego, CA, USA). For long‐read sequencing, high‐molecular‐weight genomic DNA was used to construct a single‐molecule real‐time (SMRT) sequencing library. The library, targeting an insert size of 15 kb, was generated through a process of damage repair, adapter ligation, and size selection, following a PCR‐free protocol. The prepared library was then sequenced on the PacBio Sequel II sequencing platform. Raw sequencing data (subreads.bam) were processed and quality‐controlled using SMRT Link v8.0 (PacBio) to generate the final set of circular consensus sequences (CCS) for downstream analysis. To achieve chromosome‐level scaffolding, an Hi‐C library was prepared. Briefly, cells were crosslinked with formaldehyde, and chromatin was digested with the DpnII restriction enzyme. Following end repair and biotinylation, the digested fragments were ligated under dilute conditions. After reversing crosslinks, the DNA was purified, sheared, and the biotin‐labeled fragments capturing chromatin interactions were enriched with streptavidin beads. The resulting library was quantified and quality‐checked using Qubit, Agilent 2100 Bioanalyzer, and qPCR before being sequenced on an Illumina NovaSeq 6000 platform.

Additionally, RNA sequencing (RNA‐seq) was performed on four tissues (leaves, flowers, stems, and roots) to support the gene annotation. Briefly, sequencing was conducted on the Nanopore PromethION platform using FLO‐MIN109 flow cells. Raw reads were then filtered with a minimum average quality score of 7 and a minimum length of 500 bp. Finally, ribosomal RNA was removed by alignment against the Silva rRNA database (https://www.arb‐silva.de). All library construction, sequencing, and data filtering were performed by Wuhan Benagen Tech Solutions Company Limited, Wuhan, China.

### 2.2 Genome Assembly

To generate a high‐quality genome assembly, PacBio long reads were first processed by trimming adapters and removing low‐quality sequences. The initial assembly was constructed using the Hifiasm software (https://github.com/chhylp123/hifiasm) with standard settings. Error correction was performed using gcpp in the SMRT link (v8.0) toolkit, and redundant sequences were filtered using Purge_haplotigs (v1.0.4) (Roach et al. 2018). For chromosome‐scale scaffolding, Hi‐C reads were processed by filtering out self‐ligation, dangling‐end, and dumped paired‐end reads using the HiC‐Pro software (Servant et al. 2015). Pseudochromosomes were then constructed using the ALLHIC (v0.9.12) software (Zhang et al. 2019), which employs an agglomerative hierarchical clustering approach. A Hi‐C heatmap was then generated using the Juicebox (Robinson et al. 2018). Lastly, the contigs were linked to 11 distinct chromosomes by 3D‐DNA (Dudchenko et al. 2017). To evaluate assembly quality, Illumina reads were aligned to the genome using BWA (v0.7.12) (Li and Durbin 2009), and completeness was assessed with BUSCO (v4.0.5) (Manni et al. 2021) based on conserved eukaryotic gene content.

### 2.3 Identification of Repeats and Gene Annotation

Transposable elements (TEs) were detected using the RepeatModeler (v2.0.3) (Flynn et al. 2020), RepeatMasker, and RepeatProteinMasker (Tarailo‐Graovac and Chen 2009). Tandem repeat elements were identified using the Tandem Repeats Finder (v4.09) (Benson 1999). For homology‐based predictions, BLASTP (Camacho et al. 2009) searches were performed against multiple databases, including GO, KEGG, Cluster of Orthologous Groups of Proteins (COG), NR database, and Swiss‐Prot protein database, with an *E*‐value threshold of 1e‐5. For transcriptome‐based gene prediction, RNA‐seq data from four tissues (leaf, flower, stem, and root) were aligned to the *A. venetum* genome using HISAT (v2.2.1) (Kim et al. 2015), and the transcripts were reconstructed using StringTie (v2.1.1) (Pertea et al. 2015). The longest open reading frames were searched for using TransDecoder (https://github.com/TransDecoder/TransDecoder). Ab initio gene prediction was conducted using Augustus software (v3.4.0) (Stanke et al. 2006). In addition, ncRNAs were annotated using tRNAscan‐SE (Lowe and Eddy 1997) for tRNAs, RNAmmer (Lagesen et al. 2007) for rRNAs, and INFERNAL (Nawrocki and Eddy 2013) for miRNAs and snRNAs, all with default parameters.

### 2.4 Phylogenetic Analysis and Comparative Genomics

The protein sequences of the longest transcripts from each gene in *A. venetum* and 13 other plant species, including *Calotropis gigantea* , *Camptotheca acuminata* , *Coffea canephora* , *Eustoma grandiflorum* , *Iochroma cyaneum* , *Lithospermum erythrorhizon* , *Olea europaea* , *Ophiorrhiza pumila*, *Oryza sativa* , *Platycodon grandiflorus* , *Rhododendron simsii*, *Solanum lycopersicoides* , and *Syringa oblata* , were used for gene family identification (Table S1). An all‐against‐all BLASTP search was performed to identify homologous relationships among protein sequences across these species, with an *E*‐value cutoff of 1e‐5, followed by gene family clustering using OrthoMCL software (Li et al. 2003).

Single‐copy orthologous protein sequences were aligned using MUSCLE (v5.1) (Edgar 2004), and a maximum‐likelihood phylogenetic tree was constructed using RAxML (v8.2.12) (Stamatakis 2014) with 1000 bootstrap replicates. Divergence times between these species were estimated using MCMCtree program in PAML (v4.9) with an approximate likelihood calculation method (Yang 2007), running 1000,000 Markov chain Monte Carlo (MCMC) iterations after a 200,000‐iteration burn‐in. Fossil calibration points were retrieved from the TimeTree database (http://www.timetree.org/). The gene families expansions and contractions across lineages were predicted by CAFÉ (v5.0) using a stochastic birth‐death model, retaining only significant changes (*p* < 0.05) after multiple testing correction (Han et al. 2013).

### 2.5 WGD Analysis

To estimate WGD and speciation events, an ‘all‐versus‐all’ BLASTP method (*E*‐value ≤ 1e‐5) was employed to detect paralogous genes within *A. venetum*, *E. grandiflorum* , *O. pumila* , and *C. canephora* , as well as orthologous genes between *A. venetum* and *E. grandiflorum* , *A. venetum* and *O. pumila* , and *A. venetum* and *C. canephora* . Syntenic blocks were identified using MCScanX (Wang et al. 2012), followed by the calculation of synonymous substitutions *K*s for all the aforementioned paralogous and orthologous gene pairs using yn00 in the PAML package.

### 2.6 Population Genomic Variation Analysis

**FIGURE 2:**
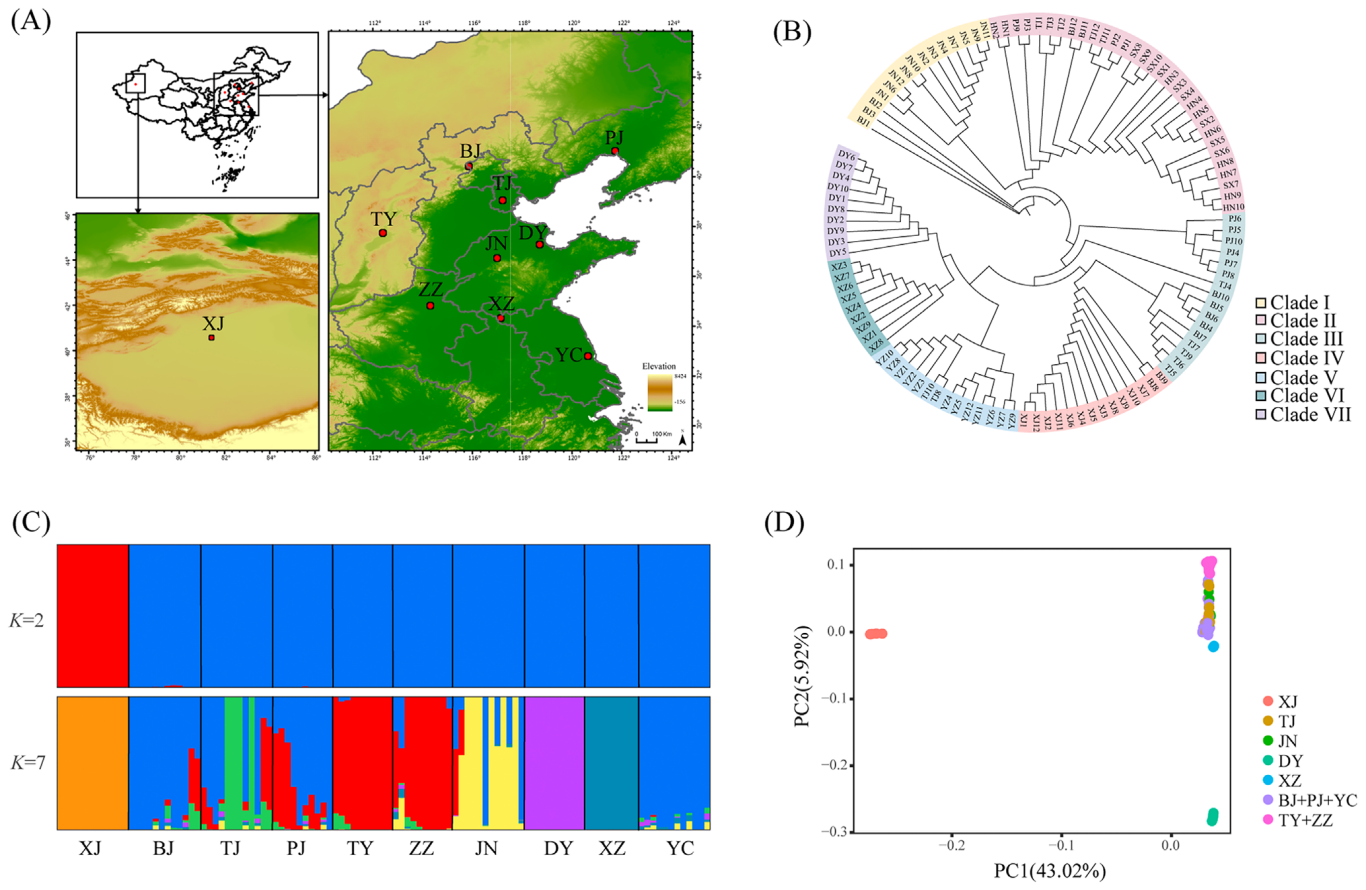
Geographic sampling, phylogeny and population genetic structure for *A. venetum*. (A) Sample locations of the 10 sampled *A. venetum* populations. (B) Neighbor‐joining phylogenetic tree of all sampled individuals. (C) Model‐based population assignment by ADMIXTURE analysis for *K* = 2 and *K* = 7. The length of each colored segment represents the proportion of the individual genome inferred from ancestral populations. (D) Principal component analysis (PCA) for all populations.

In total, 109 individuals from 10 natural populations across the distribution range of the species were sampled (Figure 2A and Table S11). Genomic DNA was isolated from leaf tissue using a modified CTAB method, followed by whole‐genome paired‐end sequencing on the BGISEQ‐500 platform to achieve ~20× genome coverage per sample. Raw reads were processed with fastp (v0.23.4) (Chen et al. 2018) to trim adapter sequences and discard low‐quality reads (Phred score < 20 and length < 50 bp). Cleaned reads were aligned to the *A. venetum* genome using BWA with standard parameters. Single nuclear polymorphism (SNP) calling was performed using GATK's HaplotypeCaller (v4.2.1) (McKenna et al. 2010) in GVCF mode, and variants were filtered using VCFtools (v0.1.16) (Danecek et al. 2011) and PLINK (Purcell et al. 2007) to retain high‐confidence SNPs. Functional annotation of variants (SNPs/InDels) was performed using ANNOVAR (Wang et al. 2010).

### 2.7 Genetic Diversity, Divergence, and Demography Analysis

Genome‐wide scans of nucleotide diversity (*π*) and inter‐population differentiation (*F*_ST_) were performed in 100‐kb nonoverlapping windows (50‐kb step size) using VCFtools. Allele frequencies, PIC, and heterozygosity of each population were calculated using the R package “snpReady” (Granato et al. 2018). A neighbor‐joining (NJ) phylogenetic tree was constructed using the PHYLIP package (Felsenstein 1989) based on the distance matrix. The population genetic structure across all individuals was inferred using ADMIXTURE (Alexander et al. 2009), with the number of clusters (*K*) ranging from 1 to 10. PCA was performed using GCTA (Yang et al. 2011) to investigate genetic structure.

The direction of gene flow among populations was inferred using Treemix (https://speciationgenomics.github.io/Treemix/). The relationship between genetic differentiation *F*_ST_/(1–*F*_ST_) and geographic distance (km) among populations was examined using Mantel tests in the R package “vegan” (Dixon 2003) with statistical significance evaluated via 999 random permutations. Demographic history was inferred using PSMC (Li and Durbin 2011) to estimate historical effective population size (*N*ₑ) fluctuations. The analysis was calibrated with a generation time of 10 years and a mutation rate of 8.0 × 10^−9^ per site per generation, which was derived from the synonymous substitution rate (*K*s) and the divergence time with a close relative (*μ* = *K*s/2T; Tajima 1989).

### 2.8 Genome‐Wide Scans for Selection Signatures and Gene Ontology Profiling

Given that the XJ region is a major habitat for the species, we sought to identify genomic regions that may have contributed to local adaptation, despite the high background divergence. To this end, we conducted genome‐wide scans for selective signatures using other populations as the reference group, interpreting the results as candidate regions that warrant further investigation. Population‐specific nucleotide diversity (*π*) and pairwise *F*_ST_ statistics were computed using VCFtools with 100‐kb sliding windows and 50‐kb step size. The top 5% of windows, based on *F*_ST_ and *π* ratio (*π*_XJ_/*π*_Others_), were considered as candidate regions under selective sweeps. Genes within these regions were extracted and analyzed for enrichment using the GO and KEGG databases to elucidate their functions.

### 2.9 Identification of Environment‐Associated Genetic Variants

Nineteen bioclimatic variables for the current period (1970–2000) were obtained from WorldClim 2.1 at a 30 arc‐seconds resolution (https://www.worldclim.org/). Ten soil variables were sourced from the Harmonized World Soil Database (v2.0) (https://www.fao.org/soils‐portal/data‐hub/soil‐maps‐and‐databases/harmonized‐world‐soil‐database‐v20). GF analysis was then performed to determine which of the 29 environmental variables best explained the distribution of genetic variation, using the R package “gradientForest” (Ellis et al. 2012). Additionally, Redundancy analysis (RDA) and partial RDA (pRDA) (Capblancq and Forester 2021) were performed to detect genotype‐environment associations using the R package vegan. Lastly, IIIVmrMLM software (Li et al. 2022) with the 3VmrMLM method was used to identify environment‐associated variants across the whole genome. The significance was determined using a Bonferroni‐corrected threshold (*p* = 0.05/m, where m denotes the number of SNPs) and LOD = 3.00.

## 3 Results

### 3.1 Genome Assembly and Annotation

A total of 25.43 Gb (108.17×) of clean data from PacBio sequencing, 37.9 Gb (161.23×) of short‐read Illumina sequencing, and 30.5Gb (129.79×) of Hi‐C paired‐end reads were generated (Table S1). *K*‐mer analysis estimated the *A. venetum* genome size to be 269.65 Mb, with a heterozygosity rate of 0.53% (Figure S1). The final assembly captured 234.73 Mb of genome sequences, with an average contig N50 of 19.11 Mb (Table 1). Approximately 96.12% (225.61 Mb) of the assembly was anchored into 11 pseudo‐chromosomes, with chromosome lengths ranging from 17.75–24.76 Mb (Table S3, Figure 1B, Figure S2). To evaluate assembly quality, we performed read‐mapping analysis using Illumina short reads, which showed a mapping rate of 99.87% and coverage of 99.96%. BUSCO analysis identified a total of 1558 single‐copy genes, accounting for 96.5% of conserved eukaryotic core genes (Table S4), demonstrating both the high accuracy and completeness in our *A. venetum* genome assembly.

**TABLE 1:** Summary of genome assembly and annotation of *A. venetum*.

Item	Number
Total_length (bp)	234,728,186.00
GC_content (%)	33.11
N50 (bp)	19,110,429.00
N90 (bp)	14,325,731.00
Average (bp)	2,794,383.17
Median (bp)	72,886.50
Min (bp)	15,339.00
Max (bp)	23,299,148.00
Pseudochromosomes	11
BUSCO completeness of assembly (%)	98.90
Repeat content (%)	39.33
Predicted protein‐coding genes	23,458.00

An integrated annotation approach revealed that 39.33% (92.33 Mb) of the genome comprised repetitive elements. Among these, long terminal repeats (LTRs) constituted nearly half of the repetitive fraction, representing 18.88% of the total genome, whereas mobile DNA elements comprised 3.11% of the genome (Table S5, Figure S3). In total, 23,458 protein‐coding genes were predicted in the *A. venetum* genome (mean gene length: 4296.67 bp; mean CDS: 1185.97 bp; 5.31 exons/gene) (Table S6). Multi‐database annotation achieved comprehensive coverage, with 95.57% of the genes receiving functional predictions through the integration of the following databases: Non‐Redundant Protein (NR) (93.67%), UniProt (93.52%), Pfam (67.1%), Gene Ontology (GO) (64.79%), and the Kyoto Encyclopedia of Genes and Genomes (KEGG) (23.23%) (Table S7). Additionally, 484 transfer RNA (tRNA), 3545 ribosomal RNA (rRNA), 572 small nuclear RNA (snRNA), and 82 microRNA (miRNA) genes were identified in the *A. venetum* genome (Table S8).

### 3.2 Phylogenetic Relationships and Whole‐Genome Duplication (WGD) Analyses

A comparative genomic analysis was conducted by comparing the *A. venetum* genome with those of 13 representative plant species. Across all 14 species, 16,251 orthologous protein families comprising 23,458 genes were identified. Comparative analysis revealed that 6560 gene families in *A. venetum* showed evolutionary conservation across species, whereas 2698 families represented lineage‐specific innovations (Table S9). Functional annotation revealed these unique genes were significantly enriched in GO categories related to nucleic acid binding and defense response (Figure S4). KEGG pathway analysis further demonstrated their significant participation in ubiquitin‐mediated proteolysis and protein processing in the endoplasmic reticulum (Figure S5).

To determine the phylogenetic position of *A. venetum* and estimate its divergence time from other lineages, we constructed a maximum‐likelihood phylogenetic tree based on 210 single‐copy orthologs from *A. venetum* and 13 other species, with *O. sativa* as an outgroup (Figure 1C). Phylogenetic analysis showed that *A. venetum* is most closely related to *C. gigantea* (Apocynaceae), with an estimated divergence time of ~30 Mya (95% highest posterior density [HPD]: 21.7–38.8 Mya). The closest relative to the Apocynaceae family is Gentianaceae, sharing a common ancestor around 58.8 Mya (95% HPD: 21.7–38.8 Mya). This estimate aligns closely with the median divergence time (~64 Mya) derived from 15 independent studies summarized in the TimeTree database (Kumar et al. 2022). The divergence of the Gentianales order was estimated to have occurred around 96.6 Mya (95% HPD: 90.6–100.1 Mya), which closely matches the median estimate (~80 Mya) for the order's origin as compiled by TimeTree.

Gene family evolution analyses revealed that 501 gene families underwent expansion, whereas 998 contracted in the *A. venetum* genome, suggesting a greater prevalence of gene family contraction during adaptive evolution (Figure 1C). KEGG enrichment analysis indicated that the significantly contracted gene families were enriched in a number of pathways, including stilbenoid, diarylheptanoid and gingerol biosynthesis (ko00945), monoterpenoid biosynthesis (ko00902), ubiquitin‐mediated proteolysis (ko04120), and the mRNA surveillance pathway (ko03015), whereas the significantly expanded gene families were functionally associated with oxidative phosphorylation (ko00190), zeatin biosynthesis (ko00908), and ribosome (ko03010) (Table S10).

Synonymous substitution rate (*K*s) analysis of *A. venetum* revealed a unimodal distribution with a clear peak at *Ks* ~ 1.7 (Figure 1D). Using the formula T = *Ks*/2λ (with λ = 8 × 10^−9^), we dated the WGD event in *A. venetum* to approximately 106 Mya. Additionally, orthologous peaks between *A. venetum* and *E. grandiflorum* (*K*s ~ 0.79) and between *A. venetum* and *C. canephora* (*K*s ~ 0.98) corresponded to divergence times of approximately 49 and 61 Mya, respectively, which were consistent with the divergence time estimates from the phylogenetic analysis.

### 3.3 Population Structure, Genetic Diversity, and Evolutionary Dynamics

We generated whole‐genome resequencing data for 109 individuals representing 10 geographically distinct populations spanning the species' eastern and northern Chinese range (Figure 2A, Table S11). After quality filtering, 96% of the reads aligned with the reference genome, providing an average sequencing depth of 29× and 95.8% genome coverage (Table S11). A total of 5,093,606 high‐quality single‐nucleotide polymorphisms (SNPs) and 1,370,653 insertions/deletions (indels) (≤ 50 bp) were identified and used for further analyses. Genetic variation annotations revealed that more than half of the variants (3,473,006) were localized in the intergenic region, followed by 19.25% (1,248,130) in the intronic region (Table S12). Among the 350,951 variants in exons, 150,098 (42.77%) were nonsynonymous mutations, and 176,176 (50.2%) were synonymous mutations. After filtering, 3,096,697 SNPs remained for subsequent population genetic analysis.

The XJ population exhibited the highest genetic diversity, with expected heterozygosity (*H*e) of 0.468, nucleotide diversity (*π*) of 0.5113 and polymorphism information content (PIC) of 0.3556, and the PJ population had the lowest values: *H*e = 0.31, *π* = 0.346 and PIC = 0.253 (Table S13). Population structure and admixture analyses strongly supported *K* = 7 as the optimal number of population clusters. XJ, TJ, DY, JN, and XZ formed distinct genetic groups, while BJ, PJ, and YC clustered together, and TY and ZZ formed another genetic group (Figure 2C, Figure S6). Phylogenetic analysis using the NJ method also distinguished seven clades (Figure 2B). Principal component analysis (PCA) revealed substantial genetic divergence, with PC1 accounting for 43.02% of total variance. This analysis distinctly separated the XJ population from all other populations, a pattern concordant with the admixture analysis at *K* = 2. The second principal component (PC2), which accounted for 5.92% of the genetic variance, differentiated the DY population from the other populations (Figure 2D).

**FIGURE 3:**
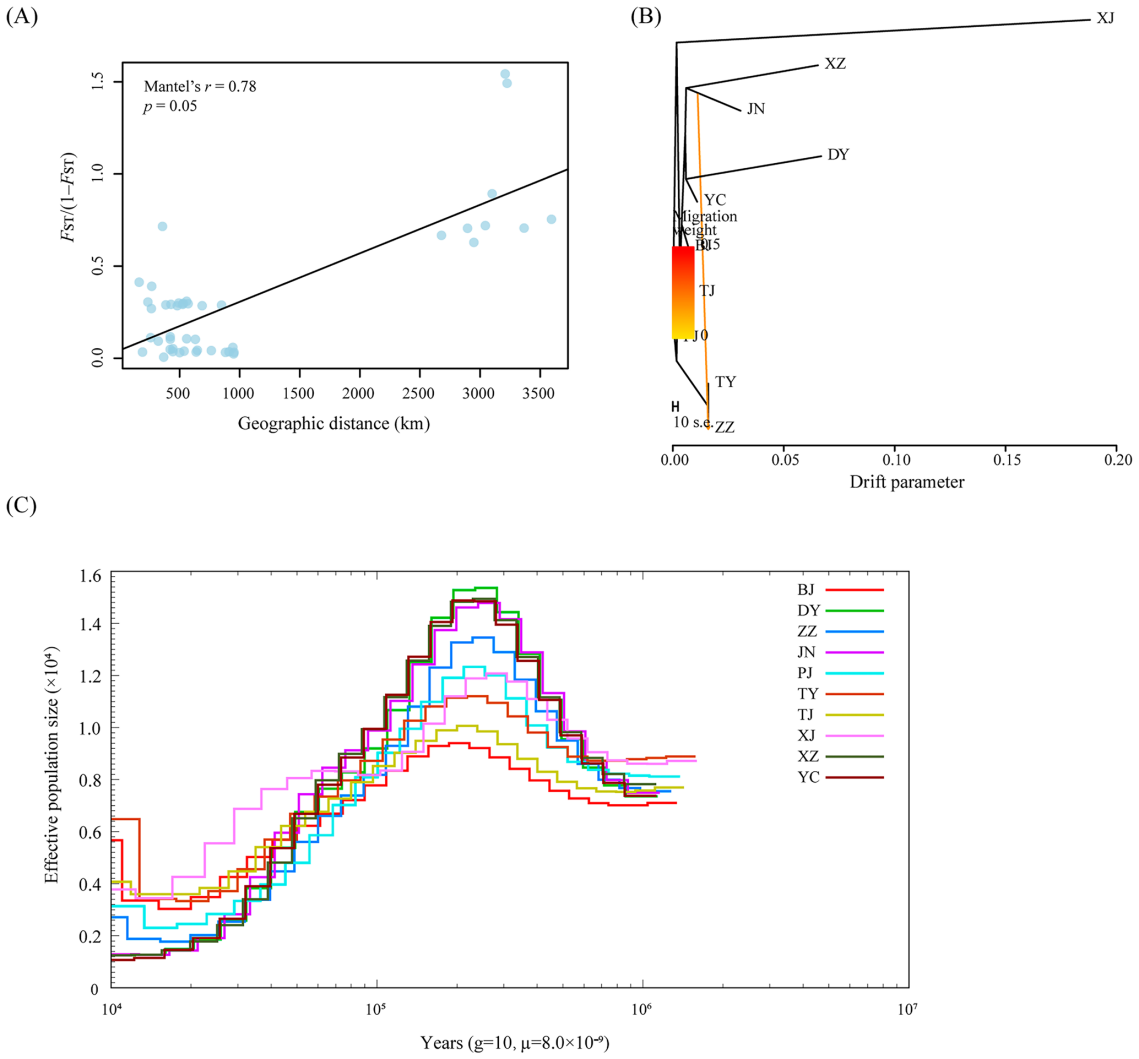
Gene flow and demographic history of *A. venetum* populations. (A) Mantel test (two‐sided) for pairwise genetic distance *F*_ST_/(1 − *F*_ST_) versus geographical distance for *A. venetum* populations. (B) Population‐based maximum‐likelihood Treemix analysis. The arrow represents the direction of gene flow. (C) Demographic history of *A. venetum* inferred by PSMC.

The pairwise *F*_ST_ values ranged from 0.006 to 0.555. The highest *F*_ST_ value was observed between XJ and XZ populations, while the lowest value was observed between ZZ and TY populations (Table S14). Notably, the pairwise *F*_ST_ values between the XJ population and all other populations were consistently high. Mantel tests confirmed a significant isolation‐by‐distance (IBD) effect, with genetic divergence (*F*_ST_/(1 − *F*_ST_)) strongly correlated with geographic distance (Mantel's *r =* 0.78, *p* = 0.05) (Figure 3A). After controlling for environmental covariates, partial Mantel analysis revealed a strong IBD pattern (Mantel's *r* = 0.81, *p* = 0.04), indicating that geographical distance was the primary driver of the population genetic structure (Table S15). TreeMix analysis, based on the highest Δ*m* value and a 99.8% variation cutoff, inferred one migration edge from the JN branch to ZZ (*m* = 1), suggesting a limited gene flow between populations (Figure 3B; Figure S7).

PSMC analysis indicated that the demographic history of *A. venetum* could be traced back to approximately 1 Mya (Figure 3C). Most populations exhibited similar patterns of historical fluctuation in *N*_e_ over time. These fluctuations suggest that the recent population decline was driven by the Last Glacial Maximum. Both the western and northern populations experienced varying degrees of expansion at the end of the last glacial period, while the eastern coastal populations (DY, JN, XZ, and YC) remained at low effective population sizes (less than 0.2 × 10^4^) (Figure 3C).

### 3.4 Genome‐Wide Detection of Selection Signatures and Functional Characterization of Adaptive Loci

**FIGURE 4:**
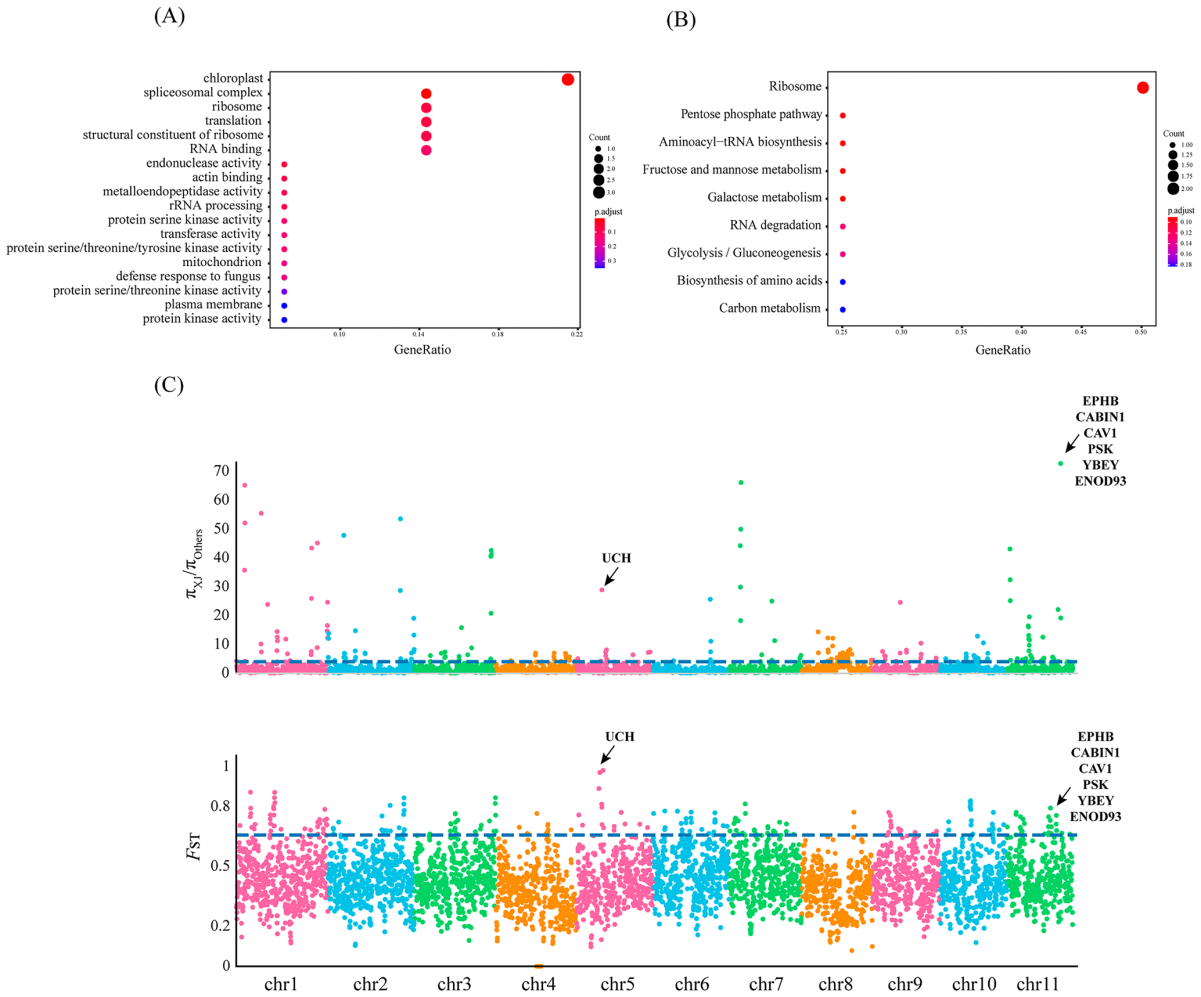
Genome‐wide selective sweeps and functional analysis of Xinjiang population of *A. venetum*. (A) GO and (B) KEGG enrichment analysis of candidate genes for strong selection. (C) Manhattan map based on *F*_ST_ and *π* ratio selected detection methods. Black arrow indicates candidate genes for strong selection.

Genetic differentiation analysis showed consistently high *F*_ST_ values between the XJ population and the other populations, indicating a high level of differentiation. Based on these results, we performed a genome‐wide selection scan for the XJ population, using the remaining populations as the reference group. Three potentially selected regions and 21 potentially selected genes were identified (Table S16). Functional enrichment analysis revealed significant associations with 18 GO terms and 9 KEGG metabolic pathways (*p* < 0.05) (Figure 4A,B). These genes displayed strong signals of selection and were mainly involved in processes such as the signal transduction pathway (EPHB, CABIN1, CAV1, and PSK), nodulation (ENOD93), and biosynthesis (YBEY) (Figure 4C).

### 3.5 Genomic Signatures of Environmental Adaptation

**FIGURE 5:**
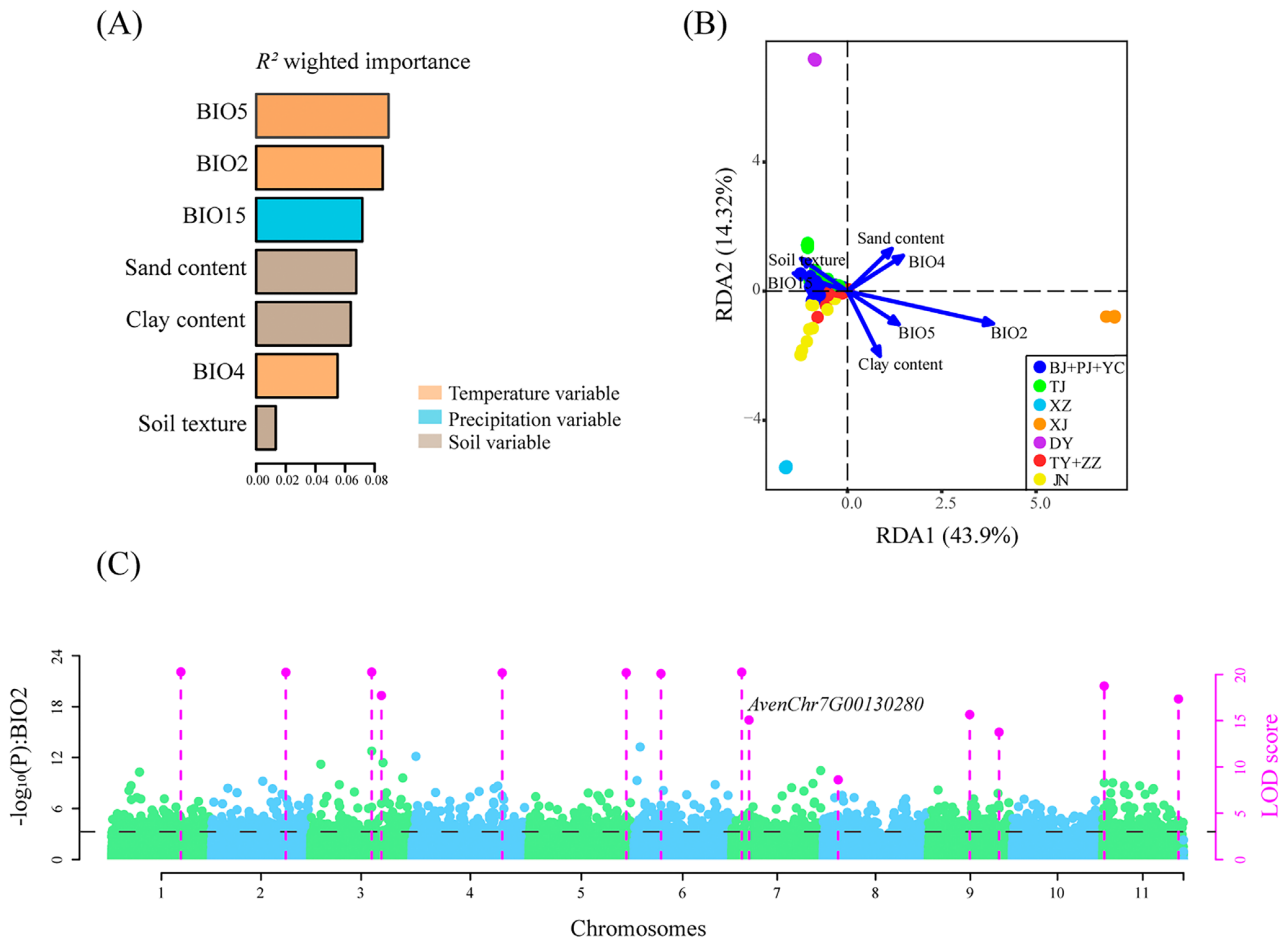
Genomic footprints of adaptation to local environmental. (A) *R* ^2^‐weighted importance of environmental variables that explain genetic gradients from gradient forest (GF) analysis. (B) Redundancy analysis of *A. venetum* populations based on on four bioclimatic factors (BIO2, mean diurnal range; BIO4, temperature seasonality; BIO5, max temperature of warmest month; BIO15, precipitation seasonality) and three soil variables (sand content, clay content, and soil texture). The Blue vectors represent environmental predictors. (C) Manhattan plot for variants associated with mean diurnal range (BIO2).

Gradient Forest (GF) analysis of 29 environmental variables identified seven uncorrelated predictors (four climatic and three soil factors; all pairwise Spearman correlations |*r*| < 0.75) suitable for redundancy analysis (RDA) analyses. The maximum temperature of warmest month (BIO5) and the mean diurnal range (BIO2) showed the highest predictive importance, followed by precipitation seasonality (BIO15), sand content, clay content, temperature seasonality (BIO4), and soil texture (Figure 5A).

The RDA revealed that 21.31% of the genetic variation among the populations could be attributed to these seven variables (*p* = 0.001). Each of the two axes explained a significant portion of the variation (RDA1: 43.9%, *p* = 0.001; RDA2: 14.32%, *p* = 0.001; Figure 5B). Although all seven environmental variables were individually significant, the most important predictor was BIO2 (7.1%, *p* = 0.001**), followed by BIO4 (3.92%, *p* = 0.001**). In contrast, soil texture contributed the least (2.41%, *p* = 0.001**) (Table 2). To assess whether these environment‐association signals were confounded by neutral population structure and IBD, we performed a partial RDA (pRDA) using geographic coordinates as conditioning variables. This analysis confirmed that spatial structure explained a substantial portion of the genetic variance (25.37%). After controlling for this spatial effect, the seven environmental variables still jointly explained a significant 29.23% of the genetic variation (*p* = 0.001) (Table S17).

**TABLE 2:** Redundancy analysis (RDA) results based on seven important environmental variables identified by GF analysis.

Environmental variables	Constrained proportion	*F*‐statistic	*p*
BIO2	0.071	8.175	0.001**
BIO4	0.039	4.372	0.001**
BIO5	0.031	3.429	0.001**
BIO15	0.032	3.592	0.001**
Sand content	0.027	3.020	0.001**
Clay content	0.029	3.200	0.001**
Soil texture	0.02406995	2.639	0.001**

*Note:* BIO2, mean diurnal range; BIO4, temperature seasonality; BIO5, max temperature of warmest month; BIO15, precipitation seasonality.

** *p* < 0.01.

Using the single environment analysis module of 3VmrMLM, we identified a total of 109 loci that were significantly associated with seven variables (Table S18). Specifically, 14, 23, 17, 19, 16, 23, 9, and 16 significant loci were found to be associated with BIO2, BIO4, BIO5, BIO15, sand content, clay content and soil texture, respectively (*p* < 1.38e‐7). The corresponding LOD scores ranged from 8.62 to 47.74 for BIO2, 22.89 to 179.47 for BIO4, 11.66 to 86.95 for BIO5, 19.16 to 73.50 for BIO15, 6.03 to 105.34 for sand, 14.41 to 89.98 for clay, and 11.11 to 84.20 for soil texture (Figure S9). The corresponding average *r* ^2^ values were 1.33%, 4.33%, 1.60%, 1.34%, 2.87%, 1.22%, and 1.32%, respectively. A total of 134 genes were identified within the 50‐kb flanking genomic region of 104 loci, with 74 loci located in the intergenic region, 35 in the genetic region, 20 in the downstream and upstream regions of the genes, and 6 within the untranslated regions (UTRs) (Table S19). Functional enrichment analysis showed that these putatively adaptive genes were partially enriched in protein binding (GO:0005515), transcription by RNA polymerase II (GO:0006366), phosphate ion transport (GO:0006817), inorganic anion transport (GO:0015698), protein dimerization activity (GO:0046983), and methyltransferase activity (GO:0008168) (Table S20).

Among the identified loci, one candidate adaptive variant (chr7:4117039) located in the exonic region of the *flavonol 4′‐sulfotransferase* (*4′‐ST*) gene (*AvenChr7G00130280*) was strongly associated with mean diurnal range (BIO2) (Figure 5C).

**FIGURE 6:**
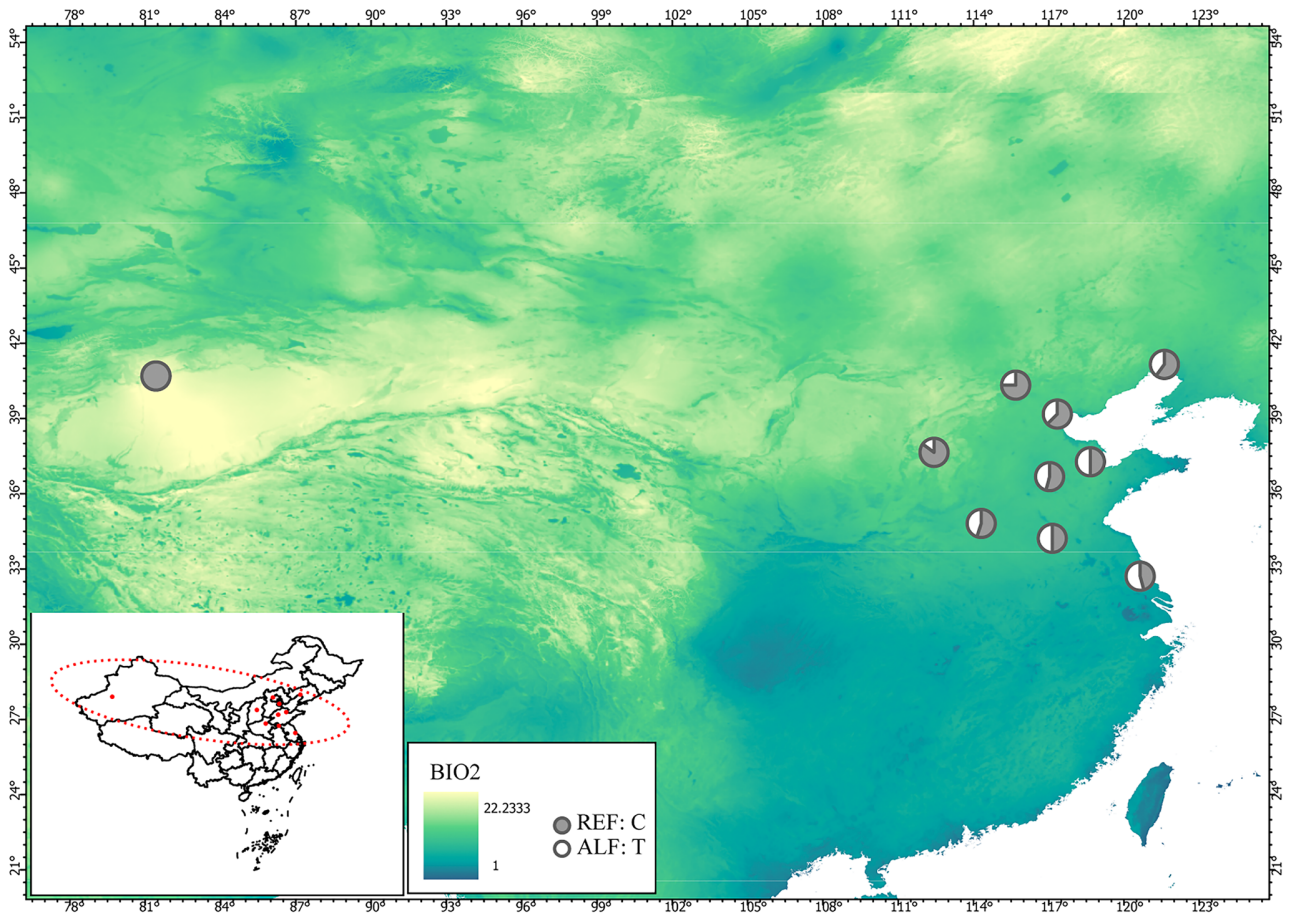
Allele frequencies of candidate adaptive SNP (chr7:4117039) associated with BIO2. The red dashed ellipse in the lower left corner indicates the distribution range of *Apocynum venetum*.

The exonic SNP exhibited distinct allele frequency clines, with the C allele predominating in northern populations (high mean diurnal range) and the T allele prevalent in southeastern populations (low mean diurnal range) (Figure 6). Genomic scans also detected additional temperature‐associated genes including *AvenChr2G00051750*, which encodes a lysine‐specific demethylase, and *AvenChr1G00017800*, an ortholog of the F‐box gene that regulates drought and salinity stress by affecting phytohormone signaling, such as abscisic acid and ethylene. In addition, a set of precipitation‐and soil component‐associated loci, including genes orthologous to *MLO*‐like, *LAC17*, and *CaM* related genes, were identified (Table S19).

## 4 Discussion

With the rapid advancement of genome sequencing technologies in recent years, research on the *A. venetum* genome has significantly expanded, encompassing the genome‐wide identification of simple sequence repeats (SSRs) and comprehensive analyses of bast fiber development and flavonoid synthesis (Li et al. 2019; Gao et al. 2023; Xiang et al. 2023). However, all previously reported *A. venetum* genomes were derived from samples collected exclusively from the Xinjiang region. Although *A. venetum* is predominantly distributed in the arid inland areas of northwest China, its range extends to the northern semi‐arid, coastal, and inland semi‐moist regions (Figure 6). The existing genomic resources do not adequately capture the genetic diversity of distinct geographical populations (Zhang et al. 2000). To address this gap, we selected a coastal semi‐humid *A. venetum* specimen and assembled its genome using PacBio HiFi sequencing to achieve higher accuracy and longer read continuity. The assembled genome exhibited a contig N50 2–5 times longer than that previously reported for *A. venetum* genomes, with a BUSCO completeness score of 98.9%. Furthermore, the number of protein‐coding genes identified in this genome surpassed those identified in previous studies, providing a more comprehensive genomic foundation for investigating *A. venetum* germplasm resources and facilitating molecular breeding for improved stress resistance.

As sessile organisms, plants rely heavily on genetic variation to adapt to changing environmental conditions (Leigh et al. 2019). Consistent with previous findings, our population genomic analysis revealed that the Xinjiang population harbored the highest genetic diversity, whereas populations in the eastern and northern coastal regions exhibited relatively low genetic diversity. Currently, Xinjiang is the largest area of production of *A. venetum* in China, with wild populations covering up to 100,000 ha in certain regions. In contrast, owing to urbanization and agricultural expansion, wild populations in the eastern and central regions, such as Shanxi, Hebei, Shandong, and Jiangsu, are relatively small and more sporadically distributed (Liu et al. 2009; Yu et al. 2024). Population genetic analysis based on SSR markers demonstrated that extant *A. venetum* populations in the coastal areas of Jiangsu Province exhibit low genetic diversity and have experienced recent bottlenecks (Yuan et al. 2020). Additionally, historical dynamic analysis suggested that the eastern coastal populations experience significant contractions during the Last Glacial Maximum. We hypothesized that historical climate change and recent anthropogenic activities have profoundly affected the population sizes of *A. venetum* in the central and eastern regions, resulting in the observed reduction in genetic diversity. Given the economic value of *A. venetum*, the loss of genetic diversity in these regions could hinder future breeding efforts to improve both fiber and medicinal traits.

Previous studies have consistently reported high genetic differentiation and limited gene flow among wild *A. venetum* populations (Peng et al. 2008; Gen 2021). Our study also detected a clear isolation‐by‐distance pattern, with gene flow detected only between the JN and ZZ populations. Both genetic clustering analysis and PCA confirmed the distinct genetic differentiation between the Xinjiang population and the other populations. Interestingly, although *A. venetum* is mainly pollinated by wind and is capable of insect pollination (Hamrick and Godt 1996), gene exchange between populations remains limited owing to the geographic isolation and reproductive characteristics of the species. The small seeds of *A. venetum* have low seed viability under field conditions, and reproduction is usually ensured through compensatory mechanisms such as clonal propagation (Chen et al. 2016; Zhao et al. 2020). Consequently, the predominance of parthenogenetic autogamy and heterogamy further hinders the gene exchange between populations. Incorporating RDA and pRDA analysis, we demonstrate that spatial structure accounts for 25.37% of total genetic variance, confirming isolation‐by‐distance as a key demographic process. Importantly, environmental factors independently explain 29.23% of genetic variance, underscoring the strength of landscape‐level local adaptation even after correcting for neutral structure. The analysis further delineated a hierarchical environmental selection regime: temperature‐related factors (BIO2, BIO4, and BIO5) dominated the primary adaptive axis (RDA1), while hydrological (BIO15) and edaphic factors emerged along a secondary axis (RDA2) once spatial covariance was removed (Figure 5B and Figure S8). These results collectively illustrate that genomic differentiation in *A. venetum* arises from the interplay of limited gene flow and multi‐layered environmental selection—responding both to macroclimatic gradients and local habitat heterogeneity across its distribution. Thus, our study advances beyond descriptive accounts of genetic structure by disentangling and quantifying the contributions of neutral and adaptive processes in driving divergence across the species' range.

Flavonoids, a phylogenetically widespread class of specialized metabolites, underpin plant adaptation through their dual roles in abiotic stress protection (e.g., antioxidant activity, UV screening) and biotic defense, with recurrent evidence for their contribution to ecological divergence (Shen et al. 2022). For instance, research has shown that the leaves of *Rhododendron* species, which grow in high‐altitude environments, are rich in flavonoids, such as quercetin and kaempferol. Several genes associated with flavonoid biosynthesis (e.g., *CHS* and *FLS*) exhibit significant allele frequency differences in high‐altitude populations, highlighting the critical role of flavonoids in helping alpine plants adapt to high UV radiation environments (Liu et al. 2022). Wu et al. (2021) quantified flavonoid components in *A. venetum* leaves from different regions and found higher levels of hyperoside and isoquercitrin in plants from the provinces of Anhui and Henan. Based on environmental genome‐wide association analysis, we identified a candidate adaptive variant in the exonic region of the *flavonol 4′‐sulfotransferases* (*4′‐ST*) gene (*AvenChr7G00130280*) that is strongly associated with mean diurnal range (BIO2) (Figure 5C). The allele frequency distribution of this adaptive SNP revealed that all individuals in the Xinjiang population carried the C allele, whereas individuals from the southeastern coastal population carried a combination of C and T alleles. Flavonol 4′‐sulfotransferase catalyzes the sulfonation modification at the 4′ position of flavonols, potentially altering their structure and enhancing their antioxidant activity, thereby aiding plant survival under oxidative stress conditions, such as high temperatures and drought (Varin et al. 1992; Daryanavard et al. 2023). Given the economic potential of *A. venetum* flavonoids in pharmaceuticals and nutraceuticals, understanding their biosynthetic regulation could facilitate the development of high‐value cultivars with enhanced flavonoid production.

Based on MaxEnt model projections, the potential suitable habitats for *A. venetum* are projected to decrease by 10% from 2041 to 2050 (Chen et al. 2023). The study also highlighted that highly suitable habitats for *A. venetum* are predominantly located in coastal areas. Global warming not only accelerates the rise of sea levels but also advances the phenophases and expands the scope of drought in the eastern coastal areas of China. These environmental pressures likely contributed to the northward migration of *A. venetum*, as a forced adaptive response to shifting habitats (Chen et al. 2023). Different *A. venetum* populations exhibit genetic specificity in stress tolerance, chemical composition, and fiber quality (Zhou et al. 2005). The extinction of any population would not only reduce the overall genetic diversity of *A. venetum* but also result in the loss of valuable traits crucial for industrial applications. Given the population sizes and levels of genetic diversity, we emphasize the urgent need for an integrated conservation strategy, including in situ conservation, seed collection, and *ex* situ conservation, particularly in eastern coastal regions.

## 5 Conclusion

In this study, we assembled a high‐quality genome of *A. venetum* that provides a valuable genetic resource for further research on this species. Through comprehensive analyses, we assessed the population structure of wild *A. venetum* populations and uncovered several loci associated with environmental adaptation, shedding light on the genetic mechanisms underlying the survival of this species in diverse habitats. These findings not only enhance our understanding of the evolutionary dynamics and adaptive potential of *A. venetum* but also provide critical insights for future conservation efforts and breeding programs. By harnessing the genetic diversity and adaptive traits of distinct populations, targeted breeding programs can be developed to enhance *A. venetum* for applications in the textile, pharmaceutical, and nutraceutical industries. Such efforts would not only meet the growing industrial demand for *A. venetum*‐derived products but also promote sustainable utilization of marginal lands.

## Conflicts of Interest

The authors declare no conflicts of interest.

## Supporting information


**Figure S1:** eva70191‐sup‐0001‐FiguresS1‐S9.docx.

**Table S1:** eva70191‐sup‐0002‐TablesS1‐S20.xlsx.

## Data Availability

The whole genome sequence data, including Illumina short reads, PacBio long reads, Hi‐C data, and transcriptome data, has been deposited on the National Genomics Data Center (NGDC, https://ngdc.cncb.ac.cn/) with the project number PRJCA038707.
