# Supplementary material for: Conservation Genomics of Apocynum venetum: Genetic Adaptation and Demographic History Across China's Saline‐Alkali Ecosystems
Source: Evol Appl. 2025 Dec 28;19(1):e70191. doi: 10.1111/eva.70191 (PMC12745339; doi:10.1111/eva.70191)
Supplement: Supplementary file 1 — Figure S1: eva70191‐sup‐0001‐FiguresS1‐S9.docx. [file EVA-19-e70191-s001.docx]

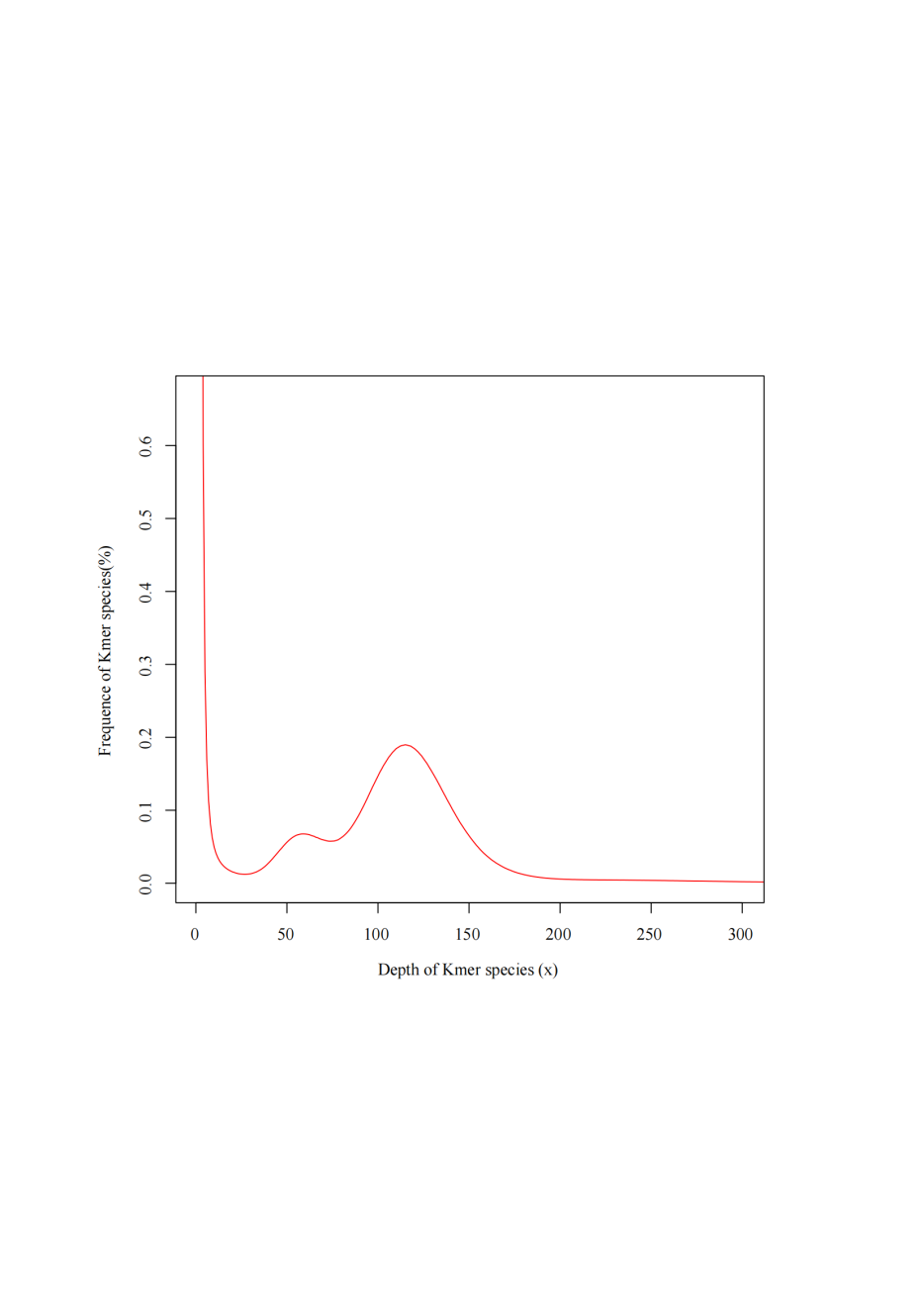


**Fig. S1.** The 19-mer analysis of *A. venetum* based on Illumina data using the GCE method.


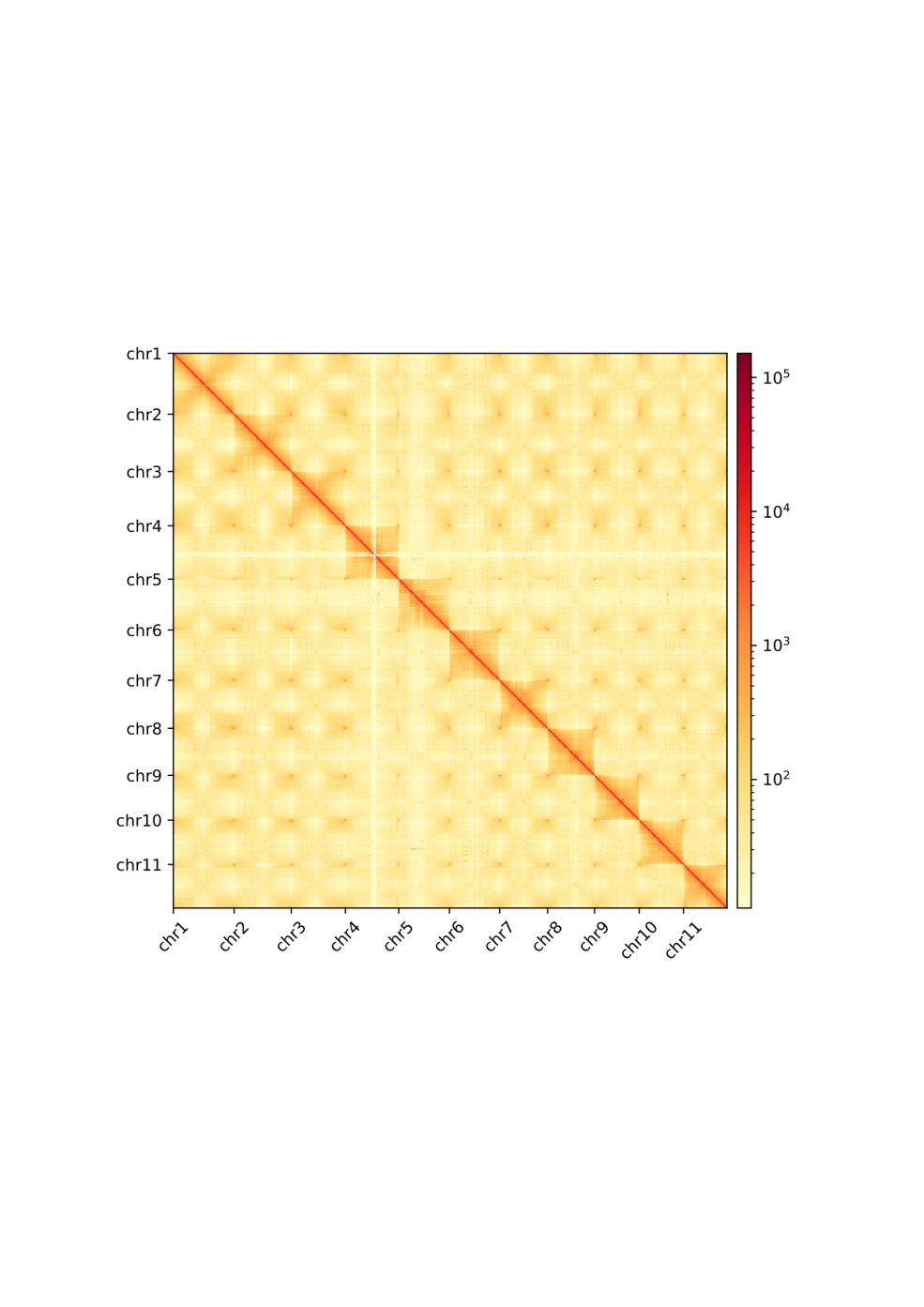


**Fig. S2.** DNA Hi-C interactions of the 11 chromosomes. Each heat map shows a normalized contact matrix, with strong contacts in red and weak contacts in yellow.


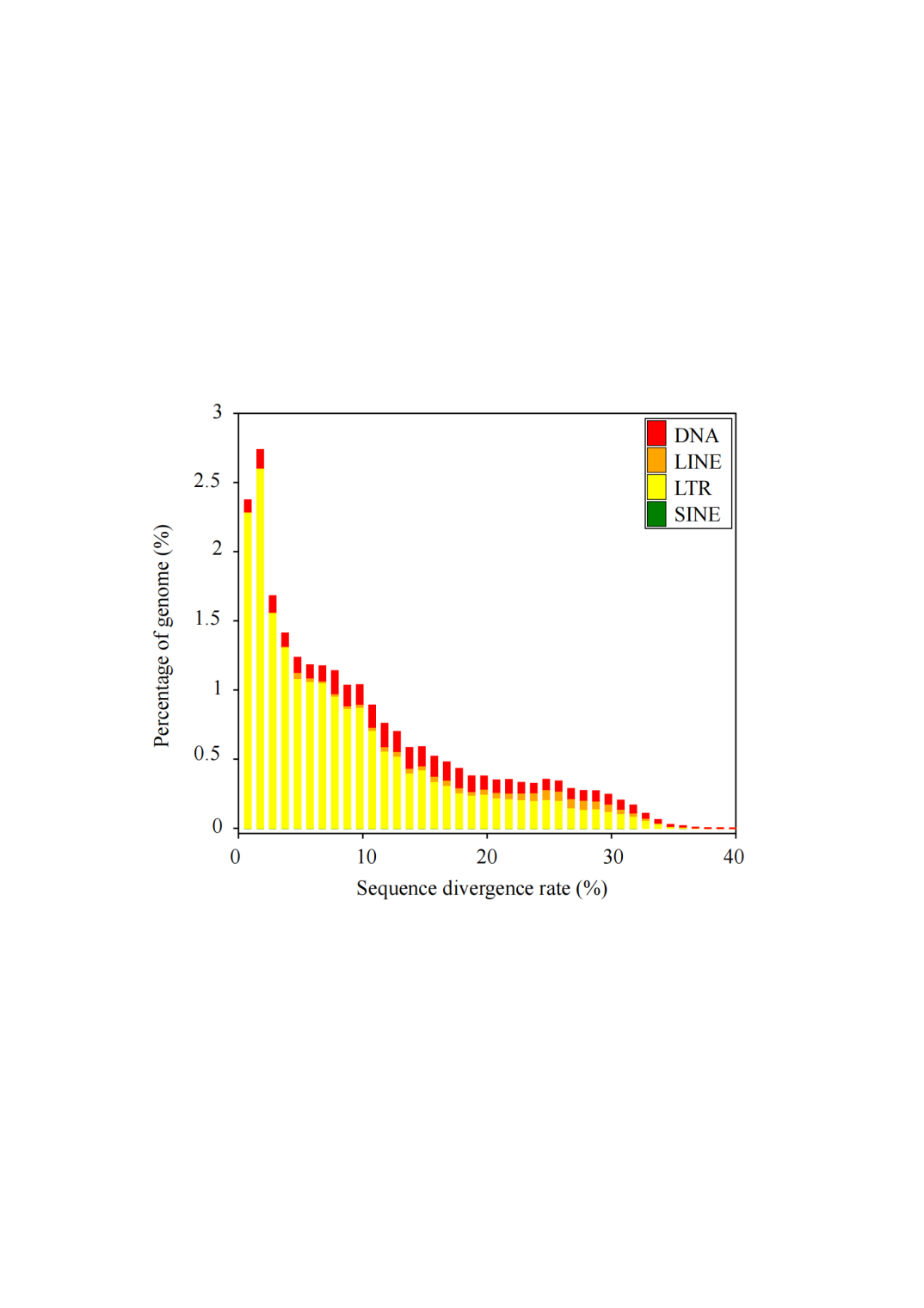


**Fig. S3.** Distribution of divergence rate of each type of transposable element (TE). The divergence rate was calculated between the identified TE elements in the genome by the de novo method. DNA: DNA transposable elements; LINE: long interspersed elements; LTR: long terminal repeats; SINE: short interspersed elements.


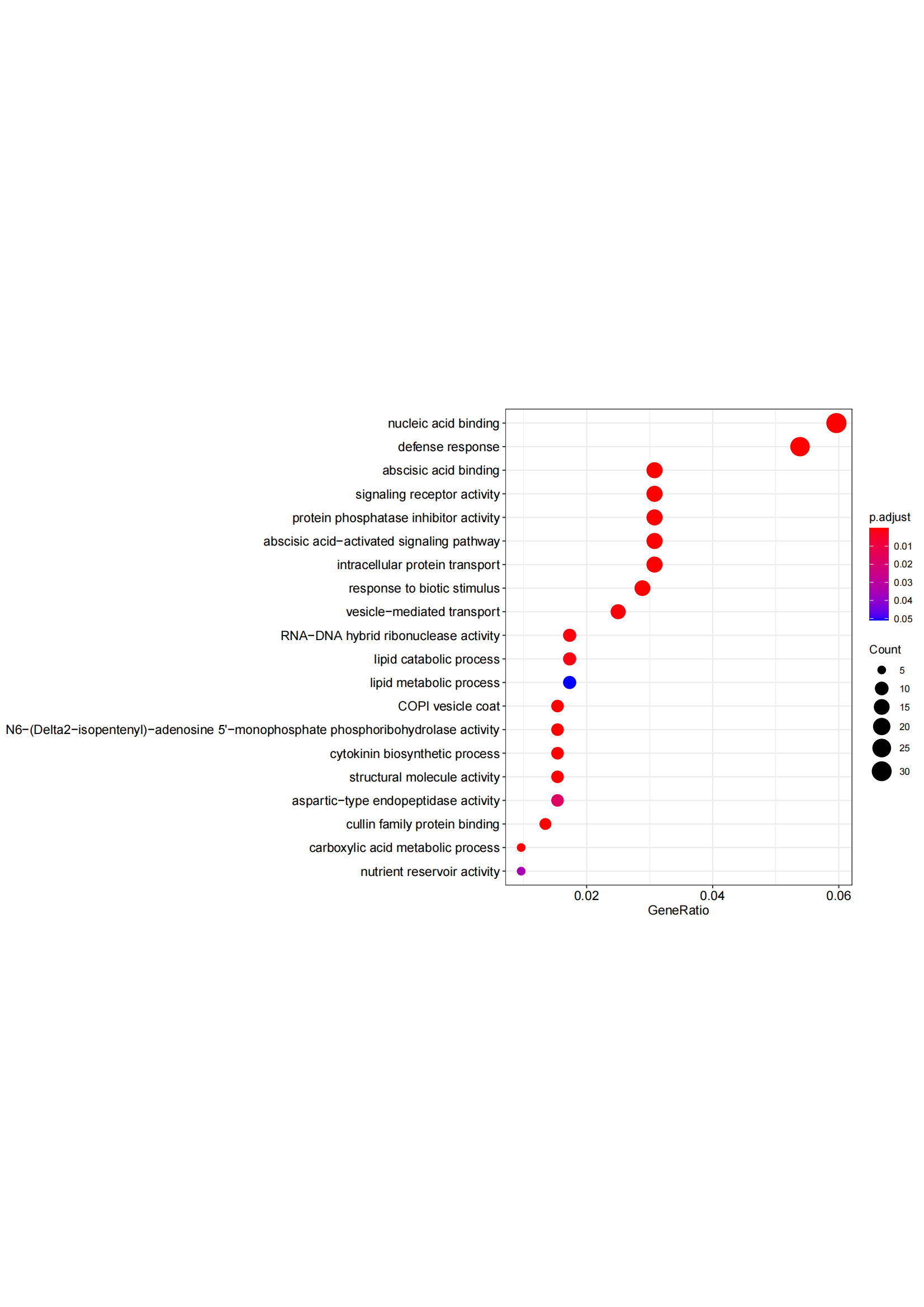


**Fig. S4.** Function enrichment analysis of specific gene families in *A. venetum*. The circle colour represents the statistical significance of enriched GO terms. The size of the circles is proportional to the number of genes in a GO term.


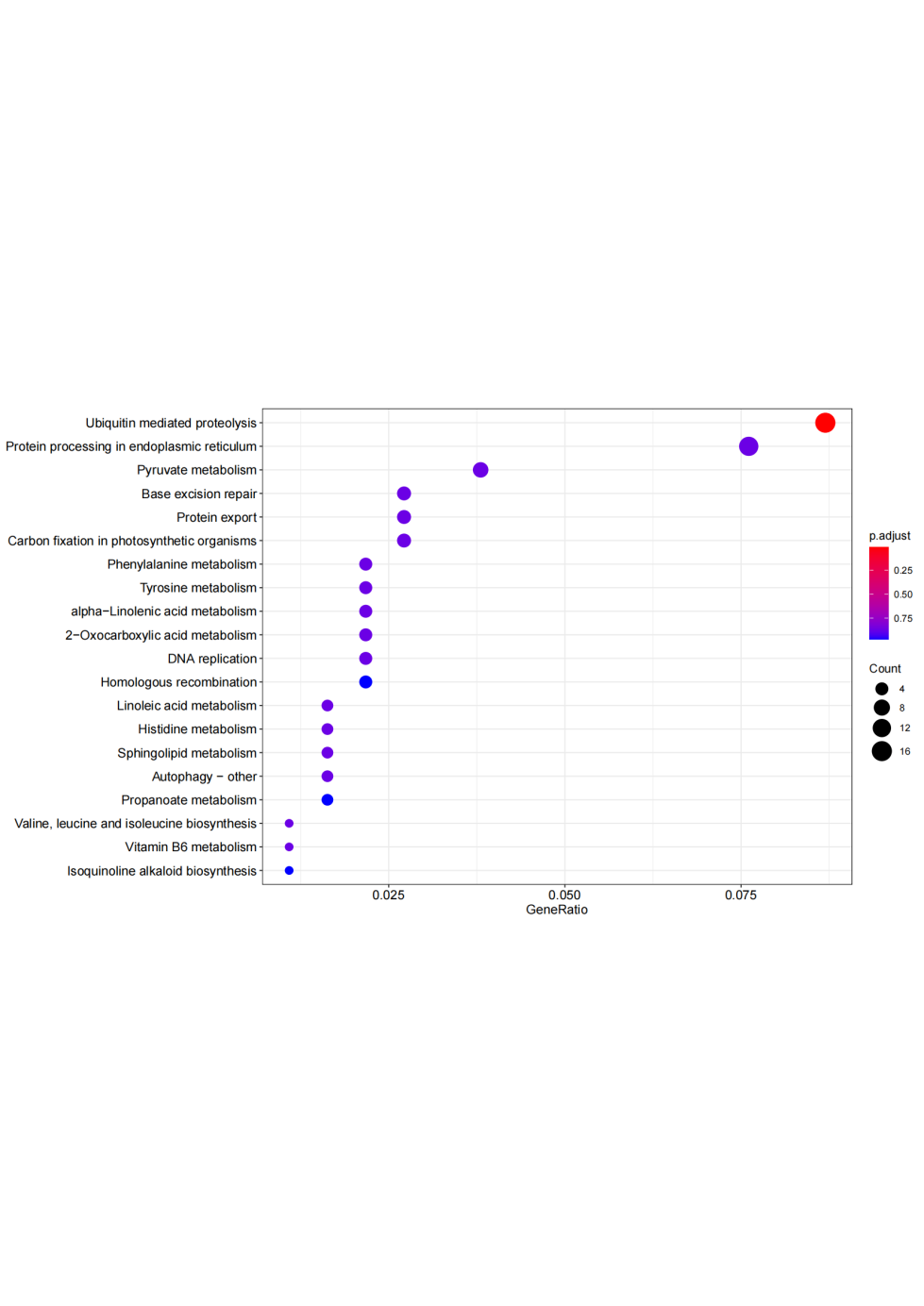


**Fig. S5.** The KEGG enrichment analysis of specific gene families in *A. venetum*. The circle colour represents the statistical significance of enriched KEGG pathways. The size of the circles is proportional to the number of genes in a KEGG pathway.


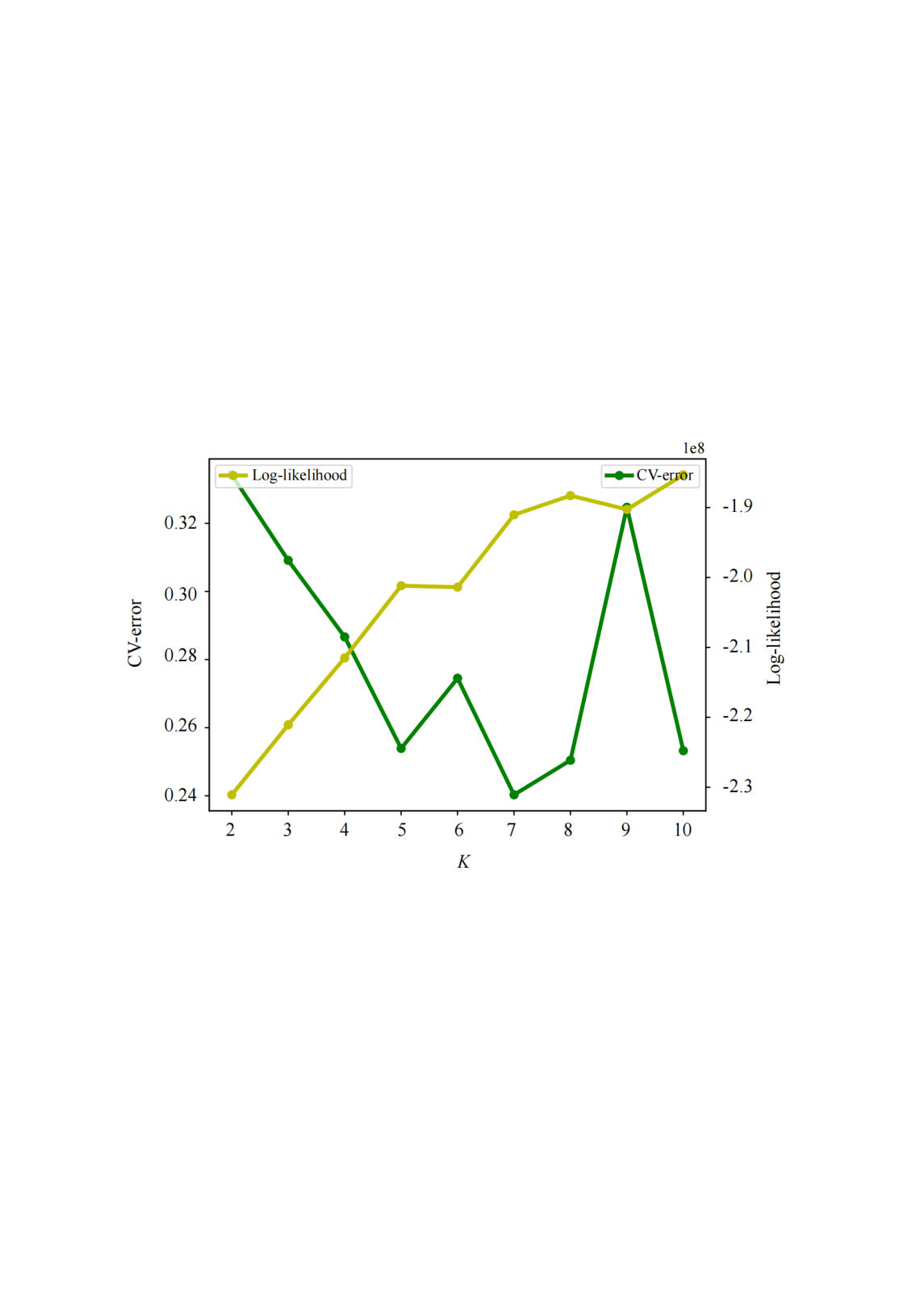


**Fig. S6.** The cross-validation (CV) error and log-likelihood distribution according to the number of clusters (*K*) by ADMIXTURE.


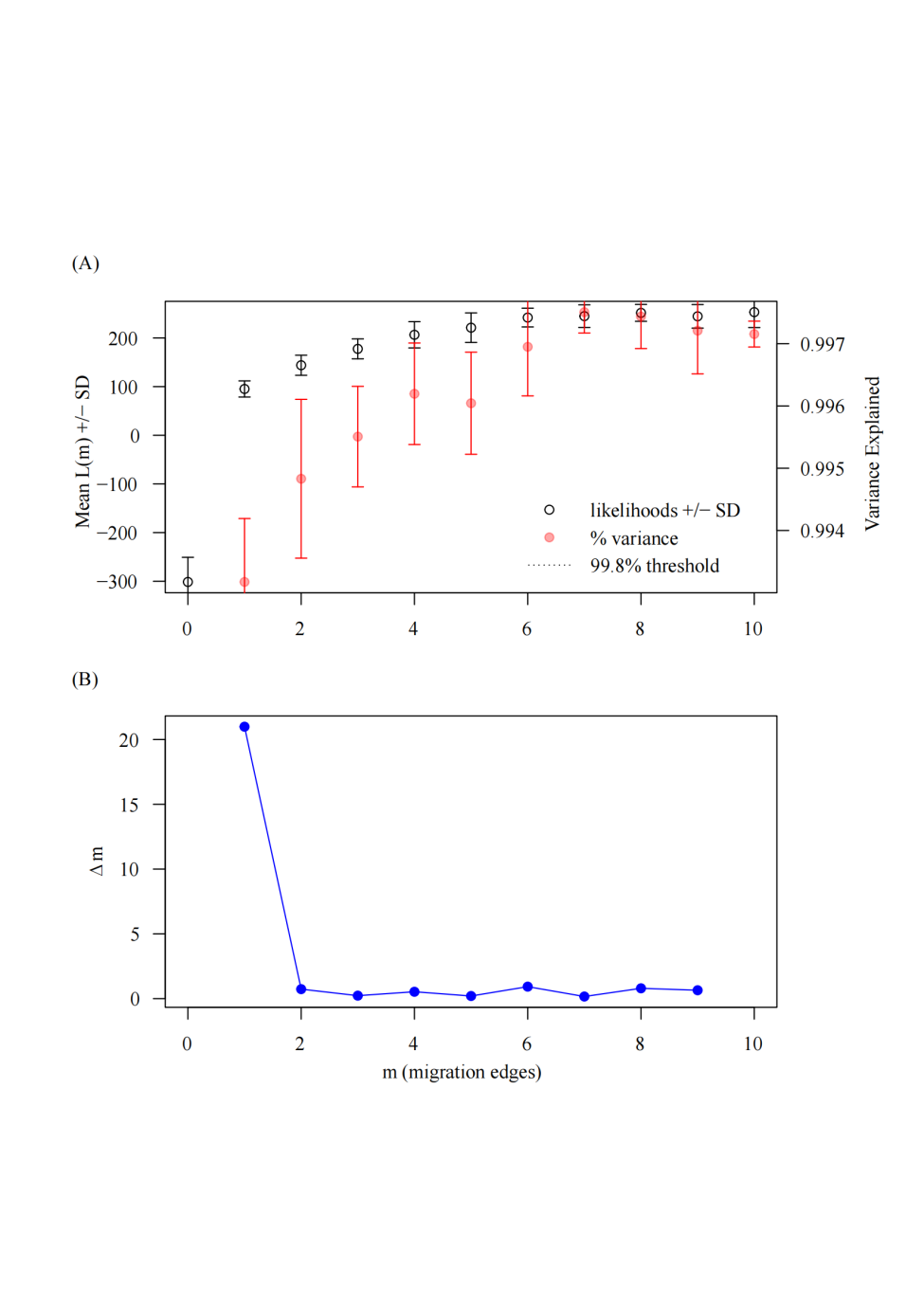


**Fig. S7.** Estimation of the optimal number of migration edges (m) for *A. venetum* populationsbased maximum likelihood tree using R package OptM. The program estimated the best m using (A) the distribution of the mean log likelihoods and % explained variance for each m and (B) an ad hoc statistic based on the second-order rate of change in the log-likelihood weighted by the standard deviation.

**
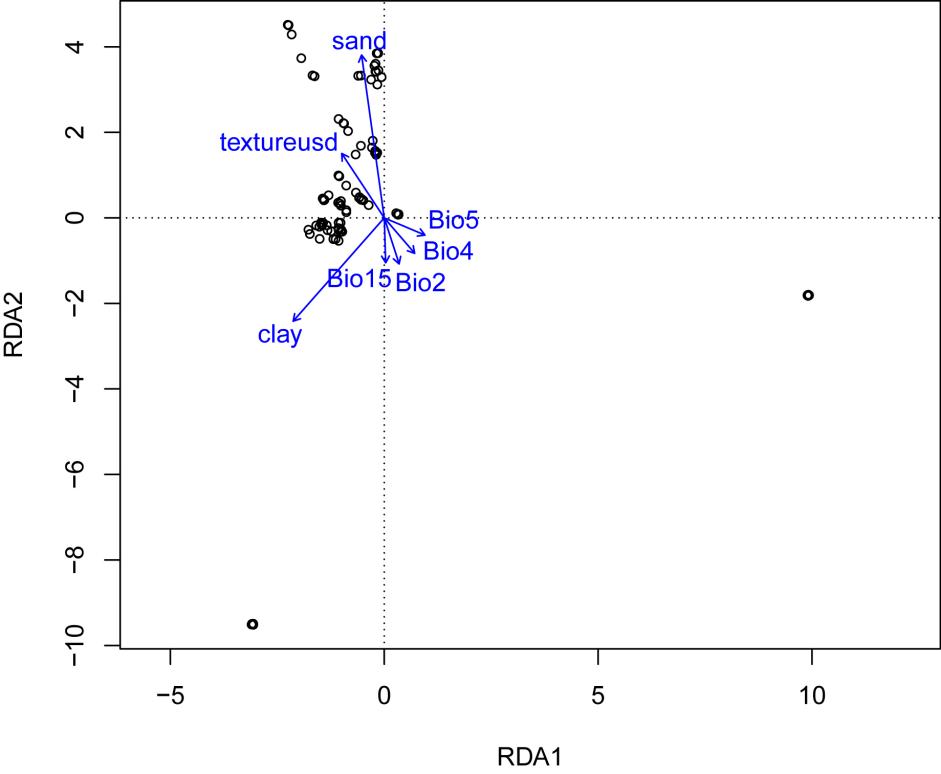
**

**Fig. S8.** Partial Redundancy analysis (pRDA) of *A. venetum* populations based on on four bioclimatic factors (BIO2: mean diurnal range; BIO4: temperature seasonality; BIO5: max temperature of warmest month; BIO15: precipitation seasonality) and three soil variables (sand content, clay content and soil texture). The Blue vectors represent environmental predictors.


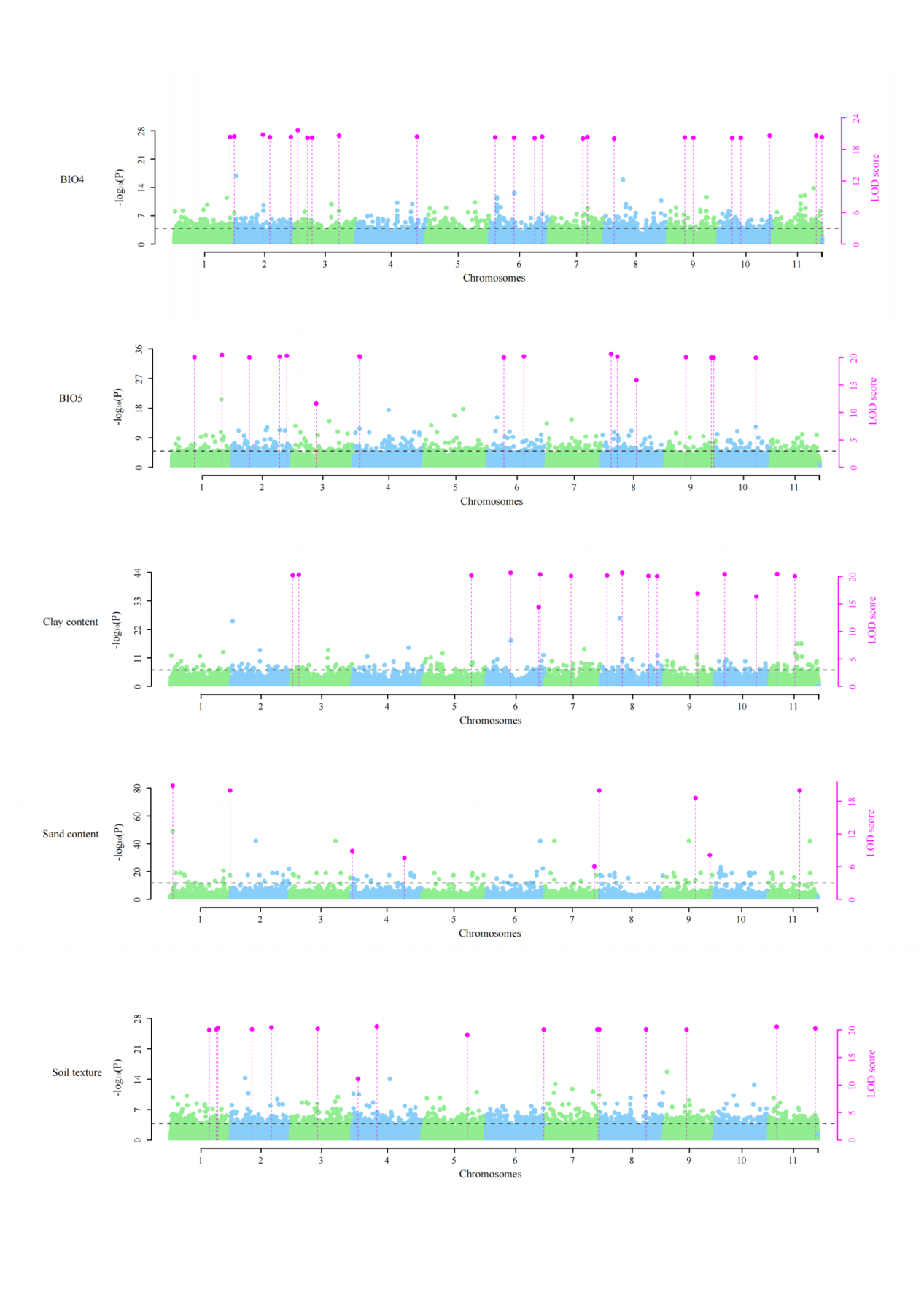
**Fig. S9.** Manhattan plot for variants associated with temperature seasonality (BIO4), max temperature of warmest month (BIO5), precipitation seasonality (BIO15), sand content, clay content, and soil texture using 3VmrMLM model. The left y-axis represents -log10 (P-values), which are calculated from single-marker genome-wide scanning based on all makers, while y-axis on the right-side represents LOD scores, which are calculated from from likelihood ratio test for QEIs, with the threshold of LOD = 3.0 (dashed line).
